# STAR in a Second: Ultra-High-Dose-Rate Spread-Out Bragg Peak Proton Therapy for Breath-Hold Stereotactic Arrhythmia Radioablation

**DOI:** 10.1016/j.adro.2026.102150

**Published:** 2026-09-11

**Authors:** Pavitra Ramesh, Marco Schwarz, Caroline Colbert, Xinyuan Claire Chen, Neil Panjwani, Stephen R. Bowen, Clemens Grassberger, Chen Wei, Uyanga Batnyam, Babak Nazer, Matthew J. Nyflot

**Affiliations:** a Department of Radiation Oncology, University of Washington Medical Center, Seattle, Washington; b Department of Radiology, University of Washington Medical Center, Seattle, Washington; c Department of Cardiology, University of Washington Medical Center, Seattle, Washington

## Abstract

**Purpose:**

Stereotactic arrhythmia radioablation (STAR) is a noninvasive treatment option for managing ventricular tachycardia (VT). Combined respiratory and cardiac motion requires management with large target volume margins or motion mitigation techniques. Techniques such as beam gating and breath-holds are limited by low-duty cycles, extending treatment times. We investigate the feasibility of ultra-high-dose-rate (UHDR) proton therapy using a spread-out Bragg peak technique with a conformal energy modulator to achieve respiratory and cardiac motion management within a single breath-hold delivery per beam for cardiac ablation, thereby reducing treatment time without compromising anatomic accuracy.

**Methods and Materials:**

Five ventricular tachycardia cases initially treated on linear accelerators with planning target volumes ranging from 118 to 254 cc were retrospectively planned with UHDR protons on an IBA Proteus Plus system with the ConformalFLASH snout. Plans were contoured on full-inspiration computed tomography phase scans to represent breath-hold anatomy and were planned for 25 Gy in 1 fraction. Plan quality metrics included target coverage (D98%, D2%, V95%) and doses to organs at risk (OARs) such as heart, stomach, and esophagus. An in-house RayStation script was developed to estimate total spot delivery time per beam to assess breath-hold feasibility. Times were validated using logfile-based analysis after delivery on the proton beamline.

**Results:**

Each plan was optimized using a combination of 2 to 5 proton fields. Target coverage was comparable with clinical photon intensity modulated radiation therapy plan metrics, demonstrating average (D98%: 2365 cGy [RBE], D2%: 2957 cGy [RBE], V95%: 98.5%). Average maximum doses to the heart, esophagus, and stomach were 3049, 631, and 894 cGy [RBE], respectively. Each field achieved high-dose rates, enabling beam delivery times ranging from 0.37 to 1.29 seconds per field.

**Conclusions:**

UHDR spread-out Bragg peak proton therapy enables conformal cardiac radioablation delivery on the order of a second per beam, which offers the opportunity to manage respiratory and cardiac motion in a single breath hold while achieving clinically acceptable plan quality.

## Introduction

Stereotactic arrhythmia radioablation (STAR) has emerged as a promising noninvasive treatment option for managing cardiac arrhythmias, particularly ventricular tachycardia (VT). Clinical studies have shown that STAR is associated with reductions in VT burden, antiarrhythmic medication use, and implantable cardioverter-defibrillator therapies, including shocks and antitachycardia pacing.^1^^,^^2^ Despite these positive outcomes, cardiac ablation radiation therapy faces unique technical challenges that significantly impact treatment planning and delivery. A primary challenge in cardiac radioablation is the combined interaction of respiratory and cardiac motion during treatment delivery, necessitating complex motion management.

Respiratory motion management is well defined in contexts such as thoracic radiation therapy and can take the form of freezing motion (eg, gating, breath hold), suppressing motion (abdominal compression), integrating motion (4-dimensional computed tomography (4DCT) average techniques), or tracking motion (multi-leaf collimator (MLC) tracking, CyberKnife).^3^, ^4^, ^5^ The Standardized Treatment and Outcome Platform for Stereotactic Therapy of Re-entrant tachychardia by a Multidiscliplinary consortium (STOPSTORM), established in 2023, found that 82% of institutions used 4DCT for respiratory assessment of STAR cases, whereas the remainder employed breath-hold techniques.^6^ A typical 4DCT average approach involves 2 to 3 mm expansion of the clinical target volume (CTV) to create an internal target volume that integrates respiratory and cardiac motion.

Cardiac-specific motion management is not routinely applied in external beam radiation therapy, but feasibility has been explored through techniques such as electrocardiogram (ECG) gating on magnetic resonance (MR)-linacs^7^ and linear accelerators.^8^ ECG gating faces practical limitations related to the duty cycle, the proportion of the motion cycle suitable for beam delivery, which is highly dependent on the patient’s heart rate for cardiac gating. Akdag et al^7^ found that an effective duty cycle of approximately 60% can be achieved at a heart rate of 47 beats per minute (bpm), but this decreases to 48% at 70 bpm and is expected to drop below 30% for heart rates above 90 bpm. For patients with VT who commonly present with elevated heart rates, these low-duty cycles may increase treatment time beyond 1 hour. Extended treatments are not well suited for patients with VT, who often cannot tolerate numerous breath-holds and may require higher acuity medical management.

Regardless of motion management approach, an additional CTV or internal target volume target expansion of 3 to 5 mm is used to create the planning target volume (PTV) to incorporate treatment uncertainty, resulting in total target expansions ranging from 2 to 8 mm.^9^, ^10^, ^11^ Variability in target volume definition, heterogeneous disease presentation,^6^ and differences in motion management contribute to wide variation in reported PTV sizes, ranging from 14 to 372 cubic centimeters (cc).^12^ Although STAR is reported as a safe alternative to techniques such as radiofrequency catheter ablations,^13^ rare but serious complications have been reported to nearby organs at risk (OARs), such as grade 4 gastric fistula resulting from proximity between the inferior cardiac surface and gastric wall.^12^ Large target expansions also increase anatomical overlap, which may increase toxicity risk or necessitate local reduction of target volume with the creation of evaluation PTVs that exclude regions overlapping with nearby OARs (often with additional planning risk volume margins). Furthermore, large margins mask the underlying problem of accurately delineating target volume, which varies widely across institutions.^6^

Ultra-high-dose-rate (UHDR) proton therapy may hold the key to safely delivering 25 Gy in a single fraction with advanced motion management. UHDR proton therapy has generated interest in recent years, primarily driven by investigations into the FLASH effect, a radiobiological effect where dose rates exceeding 40 Gy/s spare normal tissues while maintaining target control. Early implementations of UHDR in the proton setting have used transmission beams to achieve the necessary dose rates,^14^ but these approaches limit the ability to deliver conformal dose distributions to complex target geometries. Conventional proton scanning techniques require sequential delivery of multiple energy layers to cover the target, but energy layer switching times significantly reduce the average dose rate. The ConformalFLASH energy modulator addresses this limitation by employing a ridge filter that modulates the beam to create conformal Bragg peak coverage while maintaining UHDR.^15^ This technique is particularly well suited for applications where speed of delivery is important, such as cardiac ablation with minimal breath-holds. Even though the first reports of possible FLASH effects in cardiac tissue are emerging,^16^^,^^17^ the aim of our investigation is not to explore the potential advantages of the FLASH effect, but to test the feasibility of very fast treatments.

The purpose of this study is to investigate the feasibility of delivering breath-hold cardiac ablation radiation therapy using UHDR proton therapy. By leveraging the ConformalFLASH ridge filter to achieve ultrafast delivery, we aim to show that conformal Bragg peak dose distributions are achievable on the order of 1 second per beam. We then validate the achievable delivery time using an IBA proton beam system in UHDR mode. This work establishes the technical feasibility of delivering conformal STAR proton plans with UHDR for VT, inviting future investigation into the interplay between UHDR and cardiac motion management strategies.

## Methods and Materials

### Patient selection and target delineation

**Figure 1:**
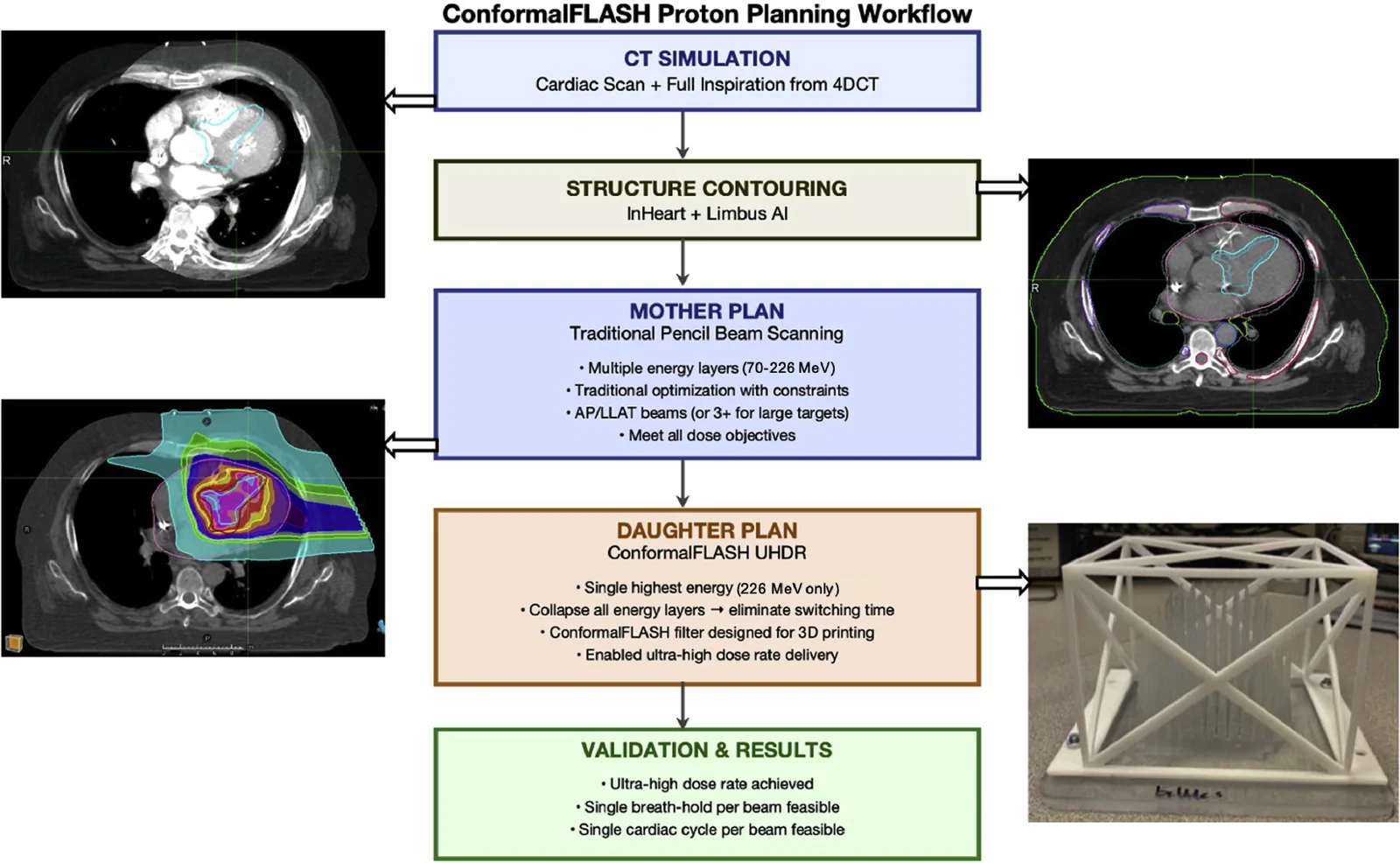
Creation of a ConformalFLASH proton daughter plan for cardiac radioablation cases. *Abbreviations:* AP/LLAT = anterior-posterior and left lateral; CT = computed tomography; UHDR = ultra-high-dose-rate.

Five consecutive cardiac radioablation cases treated at the University of Washington between 2023 and 2025 were retrospectively selected with institutional review board approval for this feasibility study. All patients had been treated with at least 1 catheter VT ablation and were originally treated with photon plans prescribing 25 Gy in a single fraction. The original clinical photon treatment plans were generated using RayStation or Ethos treatment planning systems and treated on either Elekta Synergy or Varian Ethos linear accelerators. Figure 1 illustrates the planning workflow, beginning with imaging and culminating in the development of proton treatment plans for the cardiac cases. All patients underwent diagnostic cardiac-gated computed tomography (CT) imaging and radiation therapy 4DCT simulation to capture respiratory motion. STAR target volumes and heart substructures were contoured using the InHeart three-dimensional segmentation algorithm and subsequently registered to the simulation 4DCT average data set.^18^ STAR target volumes were predominantly defined by targeting scar substrate.

All study data were anonymized. Ethos plans were exported to Raystation via script. For UHDR treatment planning, target structures were transferred from the average CT (on which the original photon plans were optimized) to the full-inspiration phase CT scan. This approach was chosen because the full-inspiration phase most closely represents the anatomy during breath-hold delivery. The average CT and full-inspiration phase CT were registered using rigid registration based on bony anatomy with manual refinement in the cardiac region. Target structures were propagated to the full-inspiration CT using the resulting transformation and subsequently reviewed by the physician. Noncardiac OARs in the chest were contoured on the full-inspiration phase scan using Limbus AI autosegmentation software (RADformation Inc), with all contours verified by the physician before treatment planning.

### Proton planning with ConformalFLASH

Proton treatment plans were generated using the RayStation 11B-FLASH treatment planning system. The ConformalFLASH planning process uses a 2-stage approach consisting of a “mother plan” and a subsequent “daughter plan.” Mother plans were developed using traditional proton pencil beam scanning techniques, such as intensity modulated proton therapy. Beam arrangements typically consisted of anterior-posterior (AP) and left lateral fields for smaller target volumes. For larger target volumes where more of the heart was encompassed, more than 2 beam angles were used to achieve adequate target coverage and normal tissue sparing.

Target coverage objectives for optimization included at least 95% of the PTV receiving 2500 cGy [RBE], at least 99% of the PTV receiving 2375 cGy [RBE], and a maximum dose (Dmax) constraint of 3200 cGy [RBE]. Selected OARs included the following constraints: heart Dmax of 3000 cGy [RBE], left ribs with no more than 1 cc receiving 2200 cGy [RBE], a stomach Dmax of 1240 cGy [RBE], and an esophagus Dmax of 1540 cGy [RBE]. Although robust optimization was not incorporated into the proton planning workflow, a robust evaluation was conducted to account for 5 mm setup uncertainties and 3% range uncertainties. This approach is used in this technical feasibility study to characterize plan stability before the implementation of full robust optimization strategies. Proton doses were calculated using a constant relative biological effectiveness (RBE) value of 1.1, consistent with the standard clinical convention. After optimization, final dose distributions were calculated for the mother plan.

On completion of the mother plan, a daughter plan was created to enable UHDR delivery. RayStation analyzes the spatial and energy-dependent fluence distribution of the mother plan to determine the cumulative spot fluence distribution across all energy layers.^19^ This is used to derive the geometry of the three-dimensional-printed patient-specific ridge filter.^20^^,^^21^ The final daughter plan dose distribution is then recalculated to verify target coverage and OAR constraints. The ridge filter attaches to the ConformalFLASH snout, which allows a maximum field size of 8 × 8 cm^2^.

### Delivery time calculation and validation

Beam delivery times were calculated using an in-house script based on our machine parameters. The model assumed a beam current of 500 nA at isocenter and a minimum spot delivery time of 1.25 ms. The script performs a time and dose analysis by calculating the time spent at each individual spot position along with the corresponding dose delivered based on the monitor units (MU) assigned to each spot. In addition to dwell times at each spot, the script accounts for transit time between consecutive spot positions. The transit times are calculated using 2 scanning speed functions (1 for each orthogonal scanning direction) derived from analysis of previous treatment delivery logfiles acquired on the IBA Proteus Plus system. This empirical approach ensures that the time calculations reflect actual machine performance characteristics. The script generates an output file reporting the total delivery time for each beam. To validate the delivery times, plans were delivered on the machine in UHDR mode and the logfiles were analyzed to confirm the feasibility of single breath-hold delivery.

We then compared calculated beam delivery times to physiological motion. Assuming a single breath hold has a duration of up to 20 seconds, the total beam delivery time for each field was calculated to determine the number of breath-holds required per beam to eliminate respiratory motion, and to estimate the number of cardiac cycles that would be needed for ECG-gated delivery.

## Results

### Patient characteristics

Patient and target characteristics for the VT cases are summarized in Table 1. CTVs ranged from 32 cc to 67 cc, with corresponding PTVs ranging from 118 cc to 254 cc. The original photon plans were typically optimized using 2 to 4 volumetric modulated arc therapy arcs, with case #1 using 9-field intensity modulated radiation therapy, whereas the daughter proton plans achieved comparable target coverage and OAR sparing using 5 or fewer proton fields per case. Larger PTVs required a greater number of proton fields to achieve full target coverage.

**Table 1:** Patient characteristics with CTV and PTV size and number of fields per photon and proton plan

Case Number	CTV size (cc)	PTV size (cc)	Number of Photon fields	Number of Proton fields
1	32	118	9 IMRT	2
2	40	129	2 VMAT	2
3	67	143	2 VMAT	2
4	57	146	4 VMAT	3
5	48	254	4 VMAT	5

*Abbreviations*: CTV = clinical target volume; IMRT = intensity modulated radiation therapy; PTV = planning target volume; VMAT = volumetric modulated arc therapy.

### Plan quality

**Figure 2:**
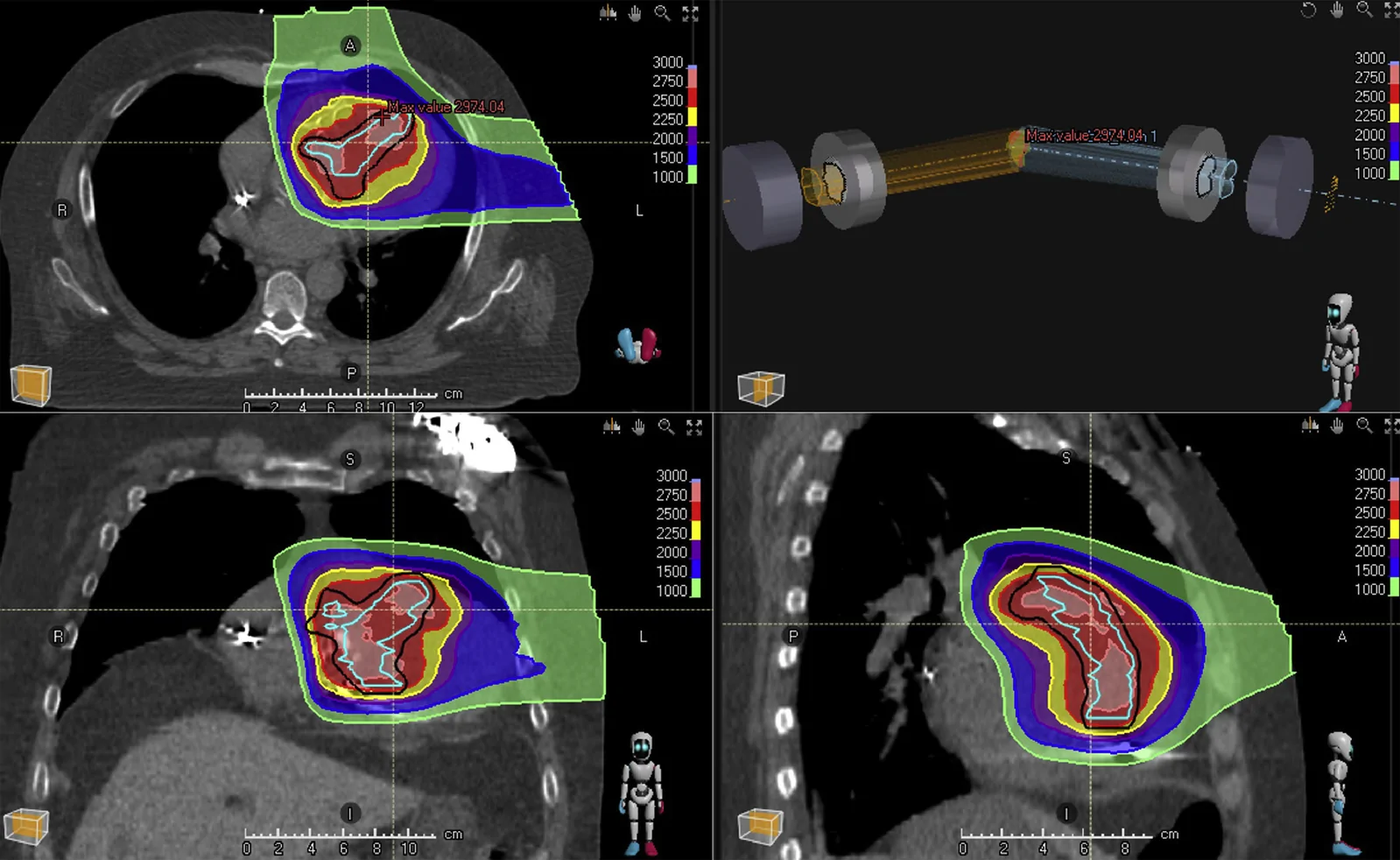
ConformalFLASH proton daughter plan for case #3. ITV is outlined in cyan, and the PTV is outlined in black. *Abbreviations:* ITV = internal target volume; PTV = planning target volume.

Figure 2 displays an example of a UHDR daughter plan for one of the VT cases. A plan quality comparison between the photon and daughter proton plans, including D98%, D50%, D2%, and V95%, as well as Dmax to the heart, esophagus, and stomach, dose to 1 cc (D1cc) of the left ribs, and mean lung dose, is included in Table 2. Across the cohort, the comparison between photon and proton UHDR plans showed some expected variations in dose metrics due to reduced lateral conformity of the ridge filter, while respecting clinical goals. Target dose metrics, as well as heart Dmax, stomach Dmax, and ribs D1cc values, were generally comparable between the 2 modalities. For proton plans, average target coverage metrics were reported as (D98%: 2365 cGy [RBE], D2%: 2957 cGy [RBE], V95%: 98.5%). Average Dmax to the heart, esophagus, and stomach were 3049, 631, and 894 cGy [RBE], respectively. Esophagus Dmax and lung Dmean demonstrated improvement with the UHDR proton plans.

**Table 2:** Mean and SD of dose metrics across 5 VT cases

Dose metric	Photon	Proton UHDR
**Target**		
D95 (cGy [RBE])	2508 ± 15	2489 ± 24
D98 (cGy [RBE])	2456 ± 20	2365 ± 66
D2 (cGy [RBE])	2814 ± 75	2957 ± 120
V95 (%)	99.5 ± 0.3	98.5 ± 1.0
**Heart-PTV**		
Mean (cGy [RBE])	990 ± 65	1284 ± 159
**Heart**		
Max (cGy [RBE])	2886 ± 110	3049 ± 156
**Esophagus**		
Max (cGy [RBE])	1014 ± 356	631 ± 565
**Stomach**		
Max (cGy [RBE])	419 ± 492	894 ± 748
**Left ribs**		
D1cc (cGy [RBE])	1601 ± 372	2072 ± 142
**Lungs**		
Mean (cGy [RBE])	243 ± 58	205 ± 12
V20 (%)	0.3 ± 0.5	6.4 ± 10.7

*Abbreviations*: PTV = planning target volume; UHDR = ultra-high-dose-rate; VT = ventricular tachycardia.

A robust evaluation in RayStation was performed assuming 5 mm setup uncertainties and 3% range uncertainties. Across the 5 VT cases, target coverage metrics remained stable, with average worst-case D95% of 2382 ± 119 cGy [RBE] and V95% of 92.2 + 2.3%. Worst-case OAR doses showed acceptable variations, with the largest relative changes observed in esophageal Dmax (868 ± 704 cGy [RBE]) and stomach Dmax (1108 ± 886 cGy [RBE]). Heart Dmax (3168 ± 161 cGy [RBE]) and mean lung dose (230 ± 12 cGy [RBE]) also met clinical goals under worst-case scenarios, despite the absence of robust optimization for ConformalFLASH plans.

### Dose rate performance and beam delivery times

**Figure 3:**
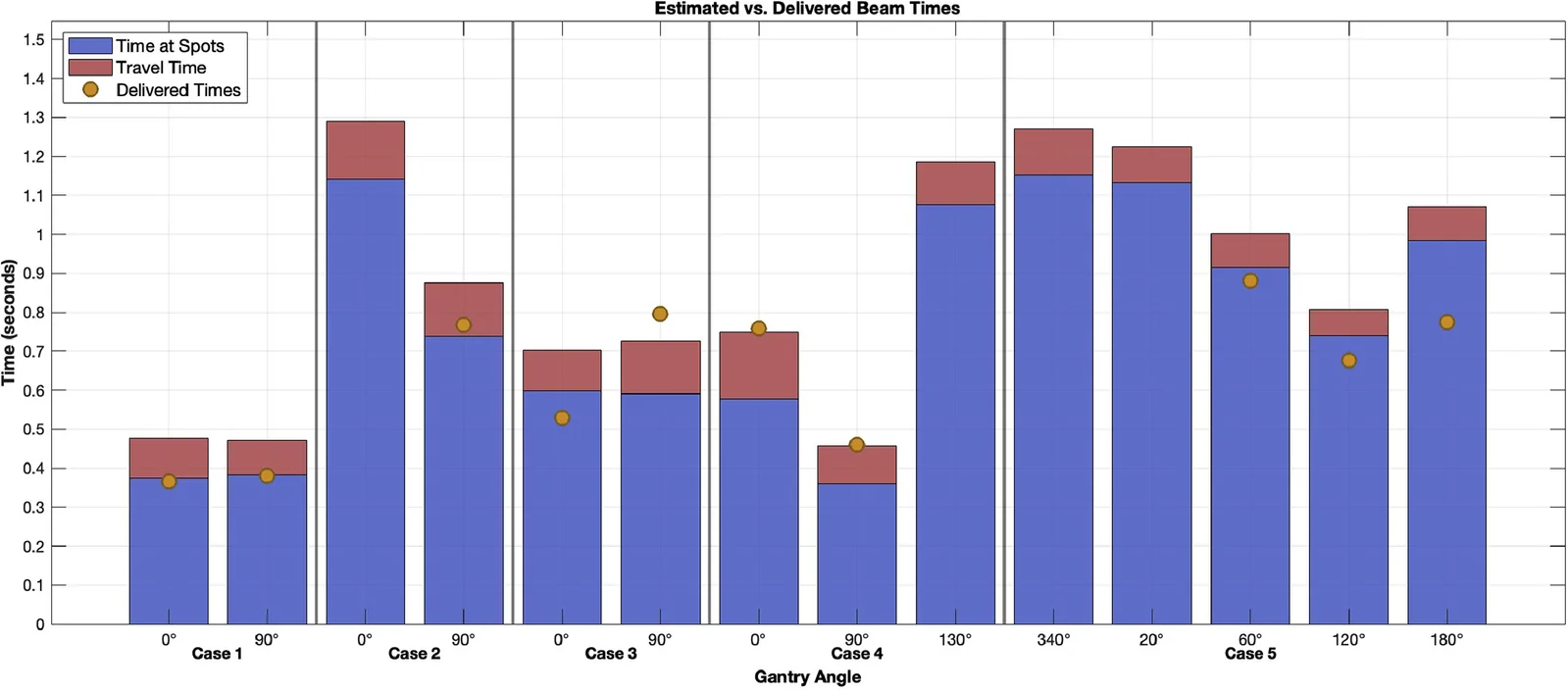
Estimated beam delivery times, comprised of time spent at spots and travel time between spots, versus actual delivery times (seconds).

Estimated and validated beam delivery times and motion management feasibility are summarized in Fig. 3. Total beam delivery times estimated from the treatment planning system ranged from 0.46 to 1.29 seconds across fields, with a mean delivery time of 0.88 ± 0.30 seconds. Ten of 14 of the beams were validated on the proton beamline, and individual beam deliveries ranged from 0.37 to 0.88 seconds. Only 10 beams were delivered because of resource limitations regarding converting the clinical beam line to a research beam line. The estimated times were generally an overpredictor of true delivery times, with an average offset of approximately 0.10 seconds. Importantly, all measured beam delivery times fall well within an acceptable range for cardiac ablation treatment in 1 breath hold.

## Discussion

This study demonstrates the feasibility of UHDR proton therapy using ConformalFLASH to deliver STAR for VT within 1 breath-hold per beam, achieving delivery times on the order of the length of the cardiac cycle. This represents a potential paradigm shift in motion management for cardiac ablation, allowing combined respiratory and cardiac management without repeated, low-duty gating cycles that can prolong treatments to multiple hours. This approach could enable margin reduction and decrease toxicity risk.

The combination of respiratory and cardiac motion means that traditional motion management strategies face significant limitations in cardiac ablation. Respiratory management techniques, such as real-time tracking of implanted pacemaker leads as surrogates during CyberKnife treatments, are relatively straightforward, although the result is extended treatment times, which are challenging for patients with VT.^22^ Cardiac motion management is substantially less developed. Recent research has explored ECG gating on MR linac^7^ and novel ultrasonographic monitoring of cardiac motion with automated interpretation of cardiac displacement in real time.^23^, ^24^, ^25^ However, these approaches have limitations due to the heart rate-dependent duty cycles and chaotic temporal interaction between cardiac and respiratory motion patterns.^26^

The demonstration that UHDR delivery could deliver STAR beams in 1 second provides technical backing for new motion management paradigms. For instance, assuming a typical breath-hold window for radiation therapy of 20 seconds and a cardiac gating duty cycle of 50% at an average heart rate of 70 bpm (0.85 cardiac cycles per second), measured delivery times of 0.37 to 0.88 seconds suggest that delivery could be completed within 1 or 2 cardiac cycles on average. For the highest MU beams, with estimated delivery times approaching 1.3 seconds, and under a conservative 30% duty cycle for elevated heart rates, the delivery of a single beam would still remain feasible within 7 cardiac cycles, or 6.5 seconds. Therefore, individual beams for each case can be plausibly delivered within a single breath hold of 20 seconds. A natural question arising from these results is how many cardiac-gated deliveries could theoretically be completed within a 20-second breath-hold, but this is highly dependent on patient comfort and gating latencies, which can extend treatment time beyond the nominal beam-on interval. Measurements reported for clinical proton systems indicate latencies of ∼200 to 250 ms,^27^ which are sufficiently small to support reasonable estimates of beam delivery time in this study, though future work will explicitly model gating interactions to quantify impacts on treatment time and target coverage.

Margin reduction is an important potential benefit to some motion management techniques, including breath hold. Margin reduction could reduce risk to gastric structures, limiting risk of severe side effects such as fistula, and obviate the need for modifications of target structures with evaluation PTV approaches. Furthermore, VT recurrence patterns may provide compelling clinical motivation for margin reduction. It is widely reported that after STAR, patients with VT may experience late VT recurrence after a period of 6 months, but in a recent study, authors reported refractory VT typically manifested proximal (64%) or distal (36%) to the original STAR treatment field rather than within it,^28^ possibly indicating development of new electrical dysfunction rather than lack of effectiveness of the radiation therapy technique. This might suggest a potential benefit to reirradiation similar to oligometastatic disease progression in spine SBRT, and the first reports of reirradiation for recurrent VT have appeared.^29^ Improved motion management and margin reduction potentially could lead to new treatment options for patients with refractory arrhythmias.

This work establishes the foundation for several important future research directions. First, ECG-gated CT simulation represents a natural extension of this work, potentially addressing the current challenges of aligning cardiac-gated CT images with average 4DCT data sets. Similarly, cardiac-gated cone beam CT in the treatment vault could enable improved target localization and verification.^30^ Finally, a FLASH radiobiological sparing effect for cardiac functionality is currently being explored.^17^ It may be possible that various tissue types involved in cardiac ablation, including cardiac muscle, gastric tissue, and lung parenchyma, may exhibit dose rate-dependent radiobiological responses. The ultrafast planning approaches developed in this study could inform future experimental investigations into potential FLASH effects in these diverse tissues adjacent to cardiac ablation targets.

It is important to note that the intention of this work is not to establish superiority of proton therapy over photon-based planning. The conformal FLASH proton plans had lower D98 and V95, higher D2, and higher mean heart-PTV and heart maximum doses, which may be explained by broadened lateral penumbra introduced by the ridge filter or fewer degrees of freedom compared to photon volumetric modulated arc therapy plans. Despite these tradeoffs, we demonstrate that PTV-based UHDR proton planning incorporating the single energy layer constraint is feasible in practice and can be achieved without requiring substantial tradeoffs in patient comfort or plan quality.

An important technical consideration for ConformalFLASH planning is the field size limitation. The snout used in this study features a maximum 8 × 8 cm^2^ field size aperture that accommodates the hedgehog filter attachment, thereby limiting the maximum deliverable field size for proton plans. For PTVs encompassing the heart, target dimensions may exceed 8 cm, which would require the use of multiple overlapping fields to cover all regions of the target, highlighting the importance of margin reduction again. Although a multifield approach introduces potential dose heterogeneity and increased total treatment time with additional fields, the aggregate delivery time remains substantially shorter than that required for conventional motion-managed techniques with multiple intensity modulated radiation therapy delivery angles or arcs, especially if employing gating duty cycles.

Another limitation of this work is that the UHDR treatment planning model is not yet validated to deliver exact treatment times. Total delivery times were reported, but dose rates were not estimated due to possible heterogeneity in dose rate. A greater understanding of dose rate might be required for future FLASH investigations. The accuracy of UHDR planning is expected to continue to improve as the utility of UHDR methods is demonstrated.

## Conclusion

This study establishes the technical feasibility of UHDR proton therapy for cardiac radioablation, demonstrating beam delivery times on the order of 1 second. By achieving delivery within a few cardiac cycles, the spread-out Bragg peak UHDR approach, in principle, circumvents duty cycle limitations inherent to conventional gating techniques, enabling efficient approaches for combined respiratory and cardiac management with clinically acceptable dosimetry for complex VT cases.

## Disclosures

None.
